# Multiple novel non-canonically transcribed sub-genomic mRNAs produced by avian coronavirus infectious bronchitis virus

**DOI:** 10.1099/jgv.0.001474

**Published:** 2020-07-28

**Authors:** Sarah Keep, Michael S. Oade, Filip Lidzbarski-Silvestre, Kirsten Bentley, Phoebe Stevenson-Leggett, Graham L. Freimanis, Chandana Tennakoon, Nicholas Sanderson, John A. Hammond, Richard C. Jones, Paul Britton, Erica Bickerton

**Affiliations:** ^1^​ The Pirbright Institute, Ash Road, Woking, GU24 0NF, UK; ^2^​ School of Biosciences, Cardiff University, Cardiff, UK; ^3^​ School of Biology, University of St Andrews, St Andrews, UK; ^4^​ Nuffield Department of Medicine, University of Oxford, Oxford, UK; ^5^​ School of Veterinary Science, University of Liverpool, Neston, UK

**Keywords:** coronavirus, IBV, transcription, sub-genomic mRNA, RNA synthesis

## Abstract

Coronavirus sub-genomic mRNA (sgmRNA) synthesis occurs via a process of discontinuous transcription involving complementary transcription regulatory sequences (TRSs), one (TRS-L) encompassing the leader sequence of the 5′ untranslated region (UTR), and the other upstream of each structural and accessory gene (TRS-B). Several coronaviruses have an ORF located between the N gene and the 3′-UTR, an area previously thought to be non-coding in the *Gammacoronavirus* infectious bronchitis virus (IBV) due to a lack of a canonical TRS-B. Here, we identify a non-canonical TRS-B allowing for a novel sgmRNA relating to this ORF to be produced in several strains of IBV: Beaudette, CR88, H120, D1466, Italy-02 and QX. Interestingly, the potential protein produced by this ORF is prematurely truncated in the Beaudette strain. A single nucleotide deletion was made in the Beaudette strain allowing for the generation of a recombinant IBV (rIBV) that had the potential to express a full-length protein. Assessment of this rIBV *in vitro* demonstrated that restoration of the full-length potential protein had no effect on viral replication. Further assessment of the Beaudette-derived RNA identified a second non-canonically transcribed sgmRNA located within gene 2. Deep sequencing analysis of allantoic fluid from Beaudette-infected embryonated eggs confirmed the presence of both the newly identified non-canonically transcribed sgmRNAs and highlighted the potential for further yet unidentified sgmRNAs. This HiSeq data, alongside the confirmation of non-canonically transcribed sgmRNAs, indicates the potential of the coronavirus genome to encode a larger repertoire of genes than has currently been identified.

## Introduction

Coronaviruses are members of the order *Nidovirales*, and possess the largest genomes of the currently identified RNA viruses. There are four genera, *Alpha-*, *Beta-*, *Gamma-* and *Deltacoronavirus*, all of which contain coronaviruses that pose a significant threat to human health, animal health and welfare, as well as food security. Notable members of the genus *Betacoronavirus* include the human pathogens severe acute respiratory syndrome coronavirus (SARS-CoV) and Middle East respiratory syndrome coronavirus (MERS-CoV), and the recent zoonotic virus SARS-CoV-2, the causative agent of COVID-19. Infectious bronchitis virus (IBV), the prototype of the genus *Gammacoronavirus*, is the aetiological agent of infectious bronchitis; an acute, highly contagious and economically important disease of poultry (reviewed elsewhere [1, 2]). As with all members of the family *Coronaviridae*, IBV possesses a large, ~27 kb, non-segmented, positive-sense, ssRNA genome, which is capped at the 5′ end and polyadenylated at the 3′ end [3]. General genome organization is shared between all coronaviruses, with the 5′ proximal two-thirds of the genome encoding 16 or, in the case of IBV, 15 non-structural proteins (nsps) as part of two large polyproteins, 1a and 1ab, with the latter produced as a result of a −1 frame shift [4, 5]. Preceding ORF 1a is an untranslated region (UTR) referred to as the 5′-UTR. The 3′ proximal end of the genome encodes the structural proteins in the order of spike (S), envelope (E), membrane (M) and nucleocapsid (N), followed by another UTR, the 3′-UTR. Interspersed within the structural genes are several small non-structural genes referred to as accessory or group-specific genes, with the location and number of these genes varying between coronaviruses. The IBV genome contains two confirmed accessory genes, gene 3 comprised of ORFs 3a, 3b and 3c (E), located between S and M, and gene 5 comprised of ORFs 5a and 5b, located between M and N, encoding accessory proteins 3a and 3b, the E protein and 5a and 5b, respectively. Gene 3 and gene 5 are each expressed from a single sub-genomic mRNA (sgmRNA). A novel group-specific protein, 4b, has additionally been proposed to be produced from a sgmRNA generated from a non-canonical transcription regulatory sequence (TRS), from a region of the genome previously referred to as the intergenic region (IR) located between gene M and gene 5 [6, 7].

Transcription of the structural and accessory genes occurs via a process of discontinuous transcription during negative-strand synthesis; a model unique to the members of the order *Nidovirales*, and first proposed by Sawicki and Sawicki in 1995 [8]. The model relies on the presence of two complementary TRSs, one located at the 5′ end of the genome, in the leader sequence, part of the 5′-UTR (TRS-L), and the second upstream of the gene, within the body of the genome (TRS-B). During minus-strand sgRNA synthesis, each TRS-B may act as a stop signal for the transcription-replication complex and facilitate a template switch through hybridization to the TRS-L. The result of this process is a nested set of negative-sense sgRNAs that act as templates for the generation of positive-sense sgmRNAs with co-terminal 5′ and 3′ ends. Each sgmRNA contains a common 5′ leader sequence and terminal 3′ sequence. With the exception of the smallest, all sgmRNAs are structurally polycistronic and possess the genetic information for several proteins. In the majority of instances, only the 5′ most proximal ORF is translated and, therefore, coronavirus sgmRNAs are functionally monocistronic, although some virus- and gene-specific exceptions do occur. In IBV, for example, gene 3 encodes proteins 3a, 3b and 3c (E), with 3a and 3b translated by leaky ribosomal scanning and 3c translated using an internal ribosomal initiation site [9].

The process of discontinuous transcription is controlled by interactions of both the TRS-L and the TRS-B. The presence of a TRS-B upstream of each structural and accessory gene is essential for the transcription of the relevant sgmRNA. The sequence of the TRSs is largely conserved across the coronavirus family and as such it is possible to use a bioinformatics approach to screen full genomic sequences to identify possible sgmRNAs and subsequent ORFs. For IBV, the proposed consensus TRS is CUUAACAA; however, there are naturally occurring variations of the TRS-B sequence suggesting flexibility and that an exact consensus sequence is not an absolute requirement for the TRS-L and TRS-B to perfectly match. For example, for the apathogenic strain Beaudette and the pathogenic strain M41, the TRS-B for the S gene is CUGAACAA. In addition, gene 5 of the M41 strain is preceded by a TRS-B of CUUAAUAA, and gene 3 of the Beaudette strain utilizes the same sequence as the S gene. Brown *et al*. have also identified variations in putative TRS-Bs of turkey coronavirus (TCoV) [10]. The matter is further complicated by the presence of functional non-canonical TRS-Bs. Bentley *et al.* identified the presence of a sgmRNA, relating to gene 4b, which is transcribed from a shortened non-canonical TRS-B of only 3 nucleotides, CAA [6]. This shortened TRS-B was found to hybridize to nucleotide positions 6, 7 and 8 of the TRS-L, with the remaining TRS in the sgmRNA derived from TRS-L. The possibility of IBV generating such transcripts was first suggested by Stern and Kennedy in 1980 [11]. Such transcripts are not unique to IBV, and have previously been described in other coronaviruses including SARS-CoV and mouse hepatitis virus (MHV) [12, 13].

Two recent publications have described the detection of a non-canonically transcribed sgmRNA in the IBV strain Beaudette, located at the very 3′ terminus of the genome, between the N gene and the poly(A) tail [7, 14]. Additionally, Dinan *et al.* identified ribosomes bound to the transcript suggesting protein translation [7]. The resulting prospective protein in the Beaudette strain, however, is prematurely truncated due to a single point mutation that results in a premature stop codon. In the present study, we similarly describe detection of a non-canonically transcribed sgmRNA correlating to the 3′ extremity of the genome across multiple strains of IBV including Beaudette, as well as TCoV. In an effort to study potential protein products of the 3′ transcript, we utilized a well-established reverse genetics system to generate two Beaudette-based rIBVs. The first rIBV has a single nucleotide deletion that removes the premature stop codon, thereby allowing a full-length polypeptide comparable to other avian coronaviruses to be produced, and the second rIBV contains a His-tag added to the N-terminus of this full-length polypeptide. In this study we also identified a gene 2-derived transcript, denoted 2*, that has previously been reported both in IBV [6] and other coronaviruses [15, 16]. We confirmed the presence of the internal gene 2 and the 3′ terminal sgmRNAs by performing deep-sequence analysis on allantoic fluid harvested from IBV-infected embryonated eggs and report on the site of leader-body fusions. Our findings highlight the potential for further unidentified non-canonically transcribed sgmRNAs produced by IBV and further demonstrate the potential for coronaviruses to encode an additional repertoire of as yet unidentified genes.

## Methods

### Cells and viruses

Primary chicken kidney (CK) cells and *ex vivo* tracheal organ cultures (TOCs) were prepared from 2 to 3-week-old specific pathogen free (SPF) Rhode Island Red (RIR) chickens as described elsewhere [17, 18]. Continuous cells lines Vero and DF1 were provided by the cell culture department at The Pirbright Institute. Embryonated RIR hens' eggs at 10 days of age were provided by the Poultry Production Unit at The Pirbright Institute.

IBV strains M41-CK [19], Beau-R [20] and Beau-CK [21], all of the Massachusetts (Mass) serotype, GI-1 lineage [22], have been described previously. IBV strain CR88 is used as a vaccine against 793B strains, also known as 4/91, which are all of the GI-13 lineage [22]. D1466, a member of the GII-1 lineage first detected in The Netherlands in the late 1970s, was a gift from Dr Franz Davelaar of the Doorn Institute, Holland [22, 23]. H120 is a widely used vaccine of the Mass serotype, GI-1 lineage [22, 24]. Italy-02, a member of the GI-21 lineage [22], originated from Italy and was a gift from Professor Elena Catelli of the University of Bologna, Italy. The QX strain of the GI-19 lineage [22] used in this study was isolated in the UK [25, 26] and has since been used to generate a vaccine [27]. All isolates of IBV and rIBV were propagated in 10-day-old RIR SPF embryonated hens’ eggs. Allantoic fluid was clarified by low-speed centrifugation at 24 to 48 h.p.i. (hours post-infection) and subsequently assayed to titrate progeny virus by plaque assay in CK cells.

### Full genome sequencing of viral stocks

As required for 454 pyrosequencing, stocks of each of CR88, D1466, H120, QX and Italy-02 were grown in order to obtain sufficient purified RNA. To generate these stocks, twenty 10-day-old embryonated SPF RIR chicken eggs per virus were infected with 100 µl IBV-infected allantoic fluid. After 24 h incubation, embryos were candled and culled by refrigeration for a minimum of 4 h. Allantoic fluid from each viral group was pooled and centrifuged to clarify supernatant.

Partial purification of IBV and isolation of viral RNA for deep sequencing were performed as previously described [28]. Briefly, IBV-infected allantoic fluid was purified by ultracentrifugation and the resulting virus pellet was resuspended in TRIzol reagent (Invitrogen, Thermo Fisher Scientific). RNA was extracted from supernatant as per the manufacturer’s instructions and quantified by NanoDrop assay. Samples were then sent to the Centre for Genomic Research (CGR), University of Liverpool, UK, for library preparation and sequencing. cDNA libraries were generated according to the GS FLX Titanium cDNA Rapid Library Preparation Method Manual [29] and sequenced using a GS FLX with Titanium series and run protocol.

The consensus sequence of the 5′ end of the genome for QX, D1466, H120, CR88 and Italy-02 was determined from RNA extracted from IBV-infected CK cells using a 5′ RACE system (Invitrogen by Life Technologies), using IBV-specific primers 5′-TGTCTGCTCACTAAAC-3′ for the reverse transcription step, and 5′-AGAACGTAGCCCAACGC-3′ for the amplification of dC-tailed cDNA step. PCR products were cloned using a TOPA TA cloning kit for sequencing (Invitrogen by Life Technologies), and the sequences of two clones were determined per virus. Sanger sequencing was performed by Source BioScience, Oxford.

Consensus sequences were assembled for each IBV strain using the mira assembler version 3.4.0.1 [30] with genome, accurate, 454 and no vector screen settings. Contigs from the initial mira assemblies were viewed, edited and ordered as necessary, based on known virus consensus sequences using the Gap4 and Gap5 programs from the Staden packages [31, 32]. The sequences were deposited in GenBank under accession numbers MN548285 for CR88, MN548286 for D1466, MN548287 for H120, MN548288 for Italy-02 and MN548289 for QX.

### IBV/rIBV infection of CK cells for intracellular RNA preparation

Confluent CK cells in six-well plates (approximately 1.2×10^6^ cells per well) were washed once with 1x PBS and inoculated with 500 µl IBV (at the highest titre possible) per well. After incubation at 37 °C for 1 h in 5 % CO_2_, the inoculum was removed and replaced with 3 ml BES (Sigma)-containing medium [33]. Infected cells were then further incubated until extensive cytopathic effect was observed, approximately 24 h.p.i., at 37 °C in 5 % CO_2_.

### IBV/rIBV infection of CK cells for assessment of viral growth kinetics

Confluent CK cells, seeded in six-well plates, were infected with either 1×10^4^ p.f.u. or 1×10^5^ p.f.u. virus and incubated at 37 °C for 1 h with 5 % CO_2_. The inoculum was removed and the cells washed twice with 1x PBS, after which 3 ml BES-containing medium was added per well. Virus-containing supernatant was harvested at 1, 24, 48, 72 and 96 h.p.i., and subsequently screened for progeny virus by titration in CK cells.

### IBV infection of *ex vivo* TOCs for assessment of intracellular RNA or ciliary activity

For the assessment of intracellular RNA, TOCs were washed once with 1x PBS and inoculated with 500 µl IBV/rIBV (at the highest titre possible) per tube in triplicate. After incubation at 37 °C for 1 h, 1 ml TOC culture medium was added [17]. Infected TOCs were incubated at 37 °C, at 8 revolutions h^−1^ for a further 23 h. For TOCs being assessed for ciliary activity as described elsewhere [34, 35], incubation was for a further 95 h, with ciliary activity assessed at 24 h intervals using an EVOS digital inverted microscope. For this assay, TOCs were infected with 1×10^5^ p.f.u. or mock infected with medium in replicates of ten.

### Preparation of RNA from CK cells, TOCs and allantoic fluid

Intracellular RNA was extracted from IBV-infected and mock-infected CK cells and *ex vivo* TOCs at 24 h.p.i. using an RNeasy mini kit (Qiagen). The manufacturer′s protocol for animal cells, spin with homogenization using a TissueLyser II (Qiagen) for 30 s at 25 Hz, was used. RNA was extracted from allantoic fluid using an RNeasy mini kit, utilizing the RNA clean-up protocol. All RNA samples were stored at −20 °C.

### Leader-body junction reverse transcription PCR (RT-PCR) analysis

Intracellular RNA was reverse transcribed using Superscript III (Life Technologies) with either virus-specific primers (Table 1) or random primer 5′-GTTTCCCAGTCACGATCNNNNNNNNNNNNNNN-3′ (Sigma). The resulting cDNA was amplified by PCR using *Taq* polymerase (Life Technologies) with either a universal forward primer (5′-CTATTACACTAGCCTTGCGC-3′) or a virus-specific primer (Table 1) located in the leader sequence of the 5′ end of the IBV genome. This was paired with either a universal primer (5′-GCTCTAACTCTATACTAGCCT-3′) or virus-specific primer (Table 1) located at the far 3′ end of the genome, directly preceding the poly(A) tail. PCRs were performed using a thermal cycler (2720 Thermal Cycler; Applied Biosystems) with the following protocol: 95 °C for 2 min; followed by 25–35 cycles of 95 °C for 30 s, 49 °C for 30 s and 72 °C for 2 min; and a final cycle of 72 °C for 10 min. PCR products were cloned using either a TOPO TA cloning kit for sequencing with a pCR4-TOPO vector or a TOPO TA cloning kit with pCR2.1-TOPO vector (both Life Technologies). One Shot MAX Efficiency TOP 10 or One Shot MAX Efficiency DH5α-T1R chemically competent cells (Life Technologies) were used for the transformation step. The bacterial colonies produced were screened either through PCR using primers M13 forward (5′-GTAAAACGACGGCCAG-3′) and M13 reverse (5′-CAGGAAACAGCTATGAC-3′) utilizing the PCR protocol described above or through *Eco*RI (Life Technologies) restriction digest of overnight cultures processed using a QIAprep spin miniprep kit (Qiagen). Both the plasmid preparations and PCR products were sequenced by Source BioScience, Oxford or GATC, Germany. Approximately 25–50 colonies from each transformation were screened.

**Table 1.** Virus-specific primers used to amplify sgmRNA in leader body junction PCR analysis The forward primer is located in the 5′ leader sequence and the reverse primer in the 3′-UTR.

Virus (strain)	Forward primer (5′→3′)	Reverse primer (5′→3′)
TCoV	CTATCATACTAGCCTTGTGC	GCTCTAACTCTATACTAGCCT
Beaudette	CTATTACACTAGCCTTGCGC	TGCTCTAACTCTATACTAGCC
M41	CTATCACACTAGCCTTGCGC	TGCTCTAACTCTATACTAGCC
H120	CTATTGCACTAGCCTTGCGC	TTCTCTAACTCTATACTAGCC
Italy-02	CATACACACTAGCCTTGTGC	GTCTCTAACTCTATACTAGCC
QX	CATACATACTAGCCTTGCGC	TGCTCTAACTCTATACTAGCC
CR88	CATACATACTAGCCTTGTGC	TGCTCTAACTCTATACTAGCC
D1466	CATACATACTAGCCTTGCGC	TTTTTTGACTCTATACTGGCC

### Northern blot analysis

mRNA was isolated from intracellular RNA extracted from CK cells at 24 h.p.i. using a Poly(A) Purist MAG kit (Ambion), following the manufacturer′s protocol. Northern blot analysis was carried out using a NothernMax-Gly kit (Ambion). Briefly, after denaturation at 50 °C with an equal volume of glyoxal loading dye for 30 min, mRNA was separated in a 1.1 % low-electroendosmosis (LE) agarose gel. The mRNA was transferred to a BrightStar-Plus positively charged nylon membrane via capillary action for 90 min and cross-linked by UV exposure (using the auto crosslink function of a Stratalinker UV Cross-linker; Stratagene). The membrane was pre-hybridized with ULTRAhyb buffer for 30 min at 42 °C, before overnight incubation at 42 °C with a DNA probe specific for the 3′ end of the genome (forward primer, 5′-CAACAGCGCCCAAAGAAG-3′, within the N gene and reverse primer located in the 3′-UTR, 5′-GCTCTAACTCTATACTAGCCT-3′). The membrane was washed and the blot developed using a BrightStar Biodetect kit (Ambion), following the manufacturer′s protocol.

### Construction of a plasmid containing modified ORF 7

The plasmids pGPT-BeauR-ORF7rep and pGPT-BeauR-ORF7rep-HIS were generated by GeneArt. Briefly, to construct pGPT-BeauR-ORF7rep, gene synthesis was carried out generating a sequence relating to Beau-R [National Center for Biotechnology Information (NCBI) GenBank accession number AY311317] genome positions 26 714–27 540 with nucleotide T27141 deleted. To construct pGPT-BeauR-ORF7rep-HIS, gene synthesis was used to generate the above fragment with nucleotides encoding a His-Tag comprised of six histidine residues inserted at position 27 114 immediately after the start codon, enabling ORF 7 to express a His-Tag on the N-terminus of the protein. This genome segment was then cloned into plasmid pGTP-NEB193, which is used as a donor molecule in the IBV reverse genetics system [20].

### Generation of a recombinant vaccinia virus containing modified IBV cDNA and the subsequent recovery of infectious IBV

The modified region of the IBV cDNA encoded in plasmids pGPT-BeauR-ORF7rep and pGPT-BeauR-ORF7rep-HIS were introduced into the full-length IBV cDNA Beau-R held within a vaccinia virus genome by homologous recombination using the transient dominant selection system described previously [33, 36, 37]. Infectious rIBVs containing the correctly modified sequence were then recovered [20, 33, 37] and passaged twice in CK cells. A stock of each rIBV was grown in 10-day-old SPF embryonated hens’ eggs, which was used for all subsequent experiments.

### Deep sequencing of Beau-CK infected allantoic fluid

Beau-CK infected allantoic fluid was harvested and clarified by low-speed centrifugation for 10 min. The resulting supernatant was then subjected to ultracentrifugation at 236 880 ***g*** for 1 h on a 30 % sucrose gradient and the pellet resuspended in 300 µl RLT buffer. Total RNA was then extracted using a Qiagen RNeasy kit, following the manufacturer′s protocol. RNA was checked for quality using a Bioanalyzer 2100 RNA Pico kit (Agilent), quantitated using a Qubit (Life Technologies) and normalized for a 100 ng input. Sequencing library preparation was performed using a NEBNext directional Ultra RNA-Seq kit (NEB). Library quality control (QC) was performed using the Bioanalyzer 2100 DNA 1000 kit and Qubit prior to pooling. The library pool was quantified using a NEBNext Illumina library quantitation kit (NEB) before being diluted and loaded onto a single lane of an Illumina HiSeq 4000.

### Analysis of deep sequencing data

Resulting reads were quality assessed using FastQC [38] with quality filtering then performed with Trim Galore! version 0.6.2 [39] using the following parameters: input as paired reads, minimum sequence length=100, quality score cut-off=30, trim 15 bp from 5′ end of R1 and R2 read. Host subtraction was performed by aligning passed quality filter (PQF) reads to the galGal6 reference genome with a *de novo* consensus sequence for Beau-CK generated from non-host derived reads using SPAdes. All quality-filtered reads were then aligned to the Beau-CK genome using BBMap [40] using default parameters. Chimeric reads were identified by initially performing an alignment with bwa [41] with reads flagged as chimeric exported and subsequently aligned using BBMap. PQF reads possessing leader sequence were identified by searching for the sequence TTAAAAATCTAGC, the reverse complement of GCTAGATTTTTAA, a sequence located within leader of Beau-CK at nucleotide positions 44–56 (NCBI GenBank accession number AY311317). To prevent duplicate matches (in forward and reverse reads), read pairs were merged using pear (Paired-End reAd mergeR) [42] prior to searching. The resulting fasta sequences were trimmed to remove the 5′ sequence upstream (relative to the viral genome) up to and including the leader search sequence and aligned to the Beau-CK reference using bwa. Coverage was reported using SAMtools (version 1.4) [43]. Data has been deposited in the Sequence Read Archive (SRA), under accession number PRJNA577406.

## Results

### Identification of a putative sgmRNA located downstream of the N gene

**Fig. 1.**
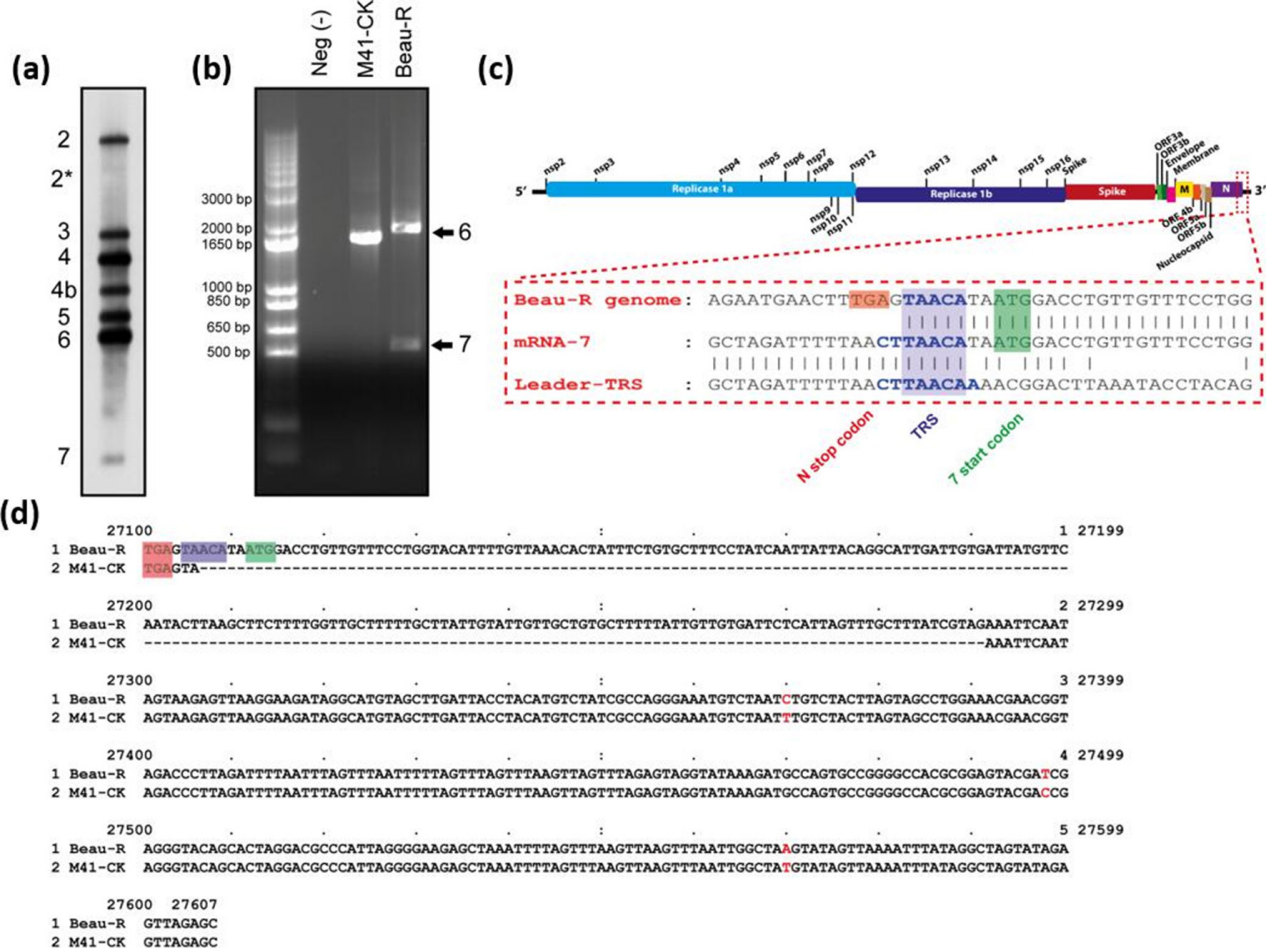
Identification of an mRNA located between N gene and the poly(A) tail for Beau-R but not M41-CK. (a) Northern blot analysis of Beau-R infected whole-cell lysate. A clearly distinguishable band, designated 7, is present at the bottom of the gel at relatively low abundance. The location of this transcript likely corresponds to the area of the genome between gene 6 (N) and the 3′-UTR. A faint band designated as 2* is also observed between genes 2 and 3. (b) Leader-body PCR analysis of Beau-R and M41-CK infected CK cell whole-cell lysate. Amplification of sgmRNAs by PCR produces two bands in the instance of Beau-R after separation by agarose gel electrophoresis. The larger sized product (~2000 bp) corresponds to the expected band size for gene 6 (N protein) transcripts. A smaller product (~500–650 bp) is also produced in Beau-R, but does not occur in M41-CK infected cell lysate. (c) Sequencing of leader-body PCR products. Sequencing of this smaller band confirms a match to the 3′ end of the virus genome (Beau-R NCBI GenBank accession number AJ311317), but the sequence also possesses a leader sequence corresponding to the 5′ end of the genome and, hence, has the characteristics of a sgmRNA. Reads do not confirm an alignment to the TRS with perfect homology and instead match only 5 nucleotides (TAACA). (d) Pairwise alignment of Beau-R and M41-CK sequences after the N stop codon. An alignment between the Beau-R and M41-CK sequences from genome position 27 100–27 607 (relative to Beau-R NCBI GenBank accession number AJ311317) reveals a 185 nucleotide deletion in M41-CK versus Beau-R, as well as three single nucleotide polymorphisms (shown in red font). The deletion includes part of the prospective TRS-B and the sgmRNA sequence. In (c) and (d), the coloured boxes indicate: red, N stop codon; blue, nucleotide matches to the canonical TRS (CTTAACA); green, prospective ORF 7 start codon.

The analysis of the full-length genome sequences of several IBV strains has identified a potential ORF at the 3′ end of the genome between the end of N and the poly(A) tail in what is considered as a hypervariable region of the IBV 3′-UTR [7, 14]. Britton and Cavanagh (in 2008) had previously considered an ORF in this area due to the presence of an AUG start codon, but discounted the possibility of another gene due to the apparent lack of a TRS-B [44]. For the IBV strain Beaudette, a canonical TRS-B of either CUUAACAA, CUGAACAA or CUUAAUAA cannot be identified in the genome between the middle of the N gene and 5′ of the putative ORF, which raised the possibility that the mechanism of transcription was either similar to that of gene 4b [6], which uses a shortened non-canonical TRS-B, or that a TRS-B of an unidentified sequence was being utilized. To determine whether a sgmRNA was produced during IBV infection that relates to the 3′ end of the genome, primary CK cells were inoculated with the rIBV Beau-R [20]. Northern blot analysis of the infected cell lysate identified a potential sgmRNA, smaller than the sgmRNA encoding the N protein (Fig. 1a). Interestingly, on a 1.1 % agarose gel this smaller putative sgmRNA runs in front of the loading dye, so it is likely that it had either run off the gel or been removed when the gel was trimmed for the RNA transfer process in previous experiments. In addition, the relatively low abundance of the mRNA compared to the other sgmRNAs produced by the structural and accessory genes raises the possibility that this band has previously been assumed to be background and the result of non-specific binding, similarly to the way 4b had previously been described [6]. In the time that this paper has been in preparation, both Dinan *et al.* (2019) [7] and An *et al.* (2019) [14] have both also identified a potential sgmRNA located between the N gene and the 3′-UTR.

### Confirmation of the presence of a sgmRNA

To confirm that the IBV-derived RNA observed on the Northern blot did relate to a novel IBV sgmRNA, intracellular RNA was extracted from CK cells infected with Beau-R or M41-CK. Leader-body junction RT-PCR was then used to amplify sgmRNAs. The primer set chosen, forward primer (5′-CTATTACACTAGCCTTGCGC-3′) located in the leader sequence and reverse primer (5′-GCTCTAACTCTATACTAGCCT-3′) located upstream of the poly(A) tail, would potentially amplify all sgmRNAs present. Due to the nature of PCRs, however, smaller templates would be preferentially amplified. A PCR product of ~2000 bp was produced from both Beau-R and M41-CK infected cells, whilst a PCR product of 500–650 bp was consistently produced from Beau-R infected cells but not those infected with M41-CK (Fig. 1b). Cloning of the Beaudette- and M41-derived PCR products, followed by sequence analysis, confirmed that the PCR products related to sgmRNAs due to the presence of the IBV leader sequence. Sequence alignments showed that the products of around 2000 bp related to the sgmRNA encoding the N gene from both M41-CK and Beau-R infected cells. The smaller PCR product only derived from Beau-R infected cells was found to align to the 3′ end of the genome, with the start of the sgmRNA immediately downstream of N (Fig. 1c). Interestingly, M41-CK has a 185 nucleotide deletion in this region of the 3′-UTR.

Sequence alignment identified that the TRS of the sgmRNA did not align with the genome with perfect homology (Fig. 1c). However, unlike gene 4b in which only 3 nucleotides (CAA) of the TRS of the sgmRNA match the genome sequence [6], 5 nucleotides (UAACA) matched in the newly identified sgmRNA. Therefore, the TRS-B was determined to consist of nucleotides UAACA, which align with positions 3 to 7 in the TRS-L (Fig. 1c). This finding supports reports by both An *et al.* (2019), who identified a TRS-B of UAACA [14], and Dinan *et al.* (2019), who identified a putative TRS-B of UAACAU [7]. Sequence alignment of the 3′-UTR for Beau-R and M41-CK identifies that the deletion seen in the latter starts 2 bp into the newly identified TRS-B (Fig. 1d), suggesting that the 5 nucleotides comprising this TRS-B are required for the transcription of the newly identified sgmRNA. Considering the identification of a functional TRS-B, and the location of the ORF that is separate to gene 6 (N gene), the newly identified sgmRNA and corresponding ORF will be referred to as gene 7.

### A sgmRNA relating to gene 7 is expressed from several IBV strains and TCoV

To establish whether the newly identified sgmRNA was specific to the Beaudette strain or was present in other strains of IBV, full genome sequencing of five strains of IBV was undertaken using 454 pyrosequencing technology. The strains investigated included vaccine strains CR88 and H120, as well as field isolates D1466, Italy-02 and QX.

**Fig. 2.**
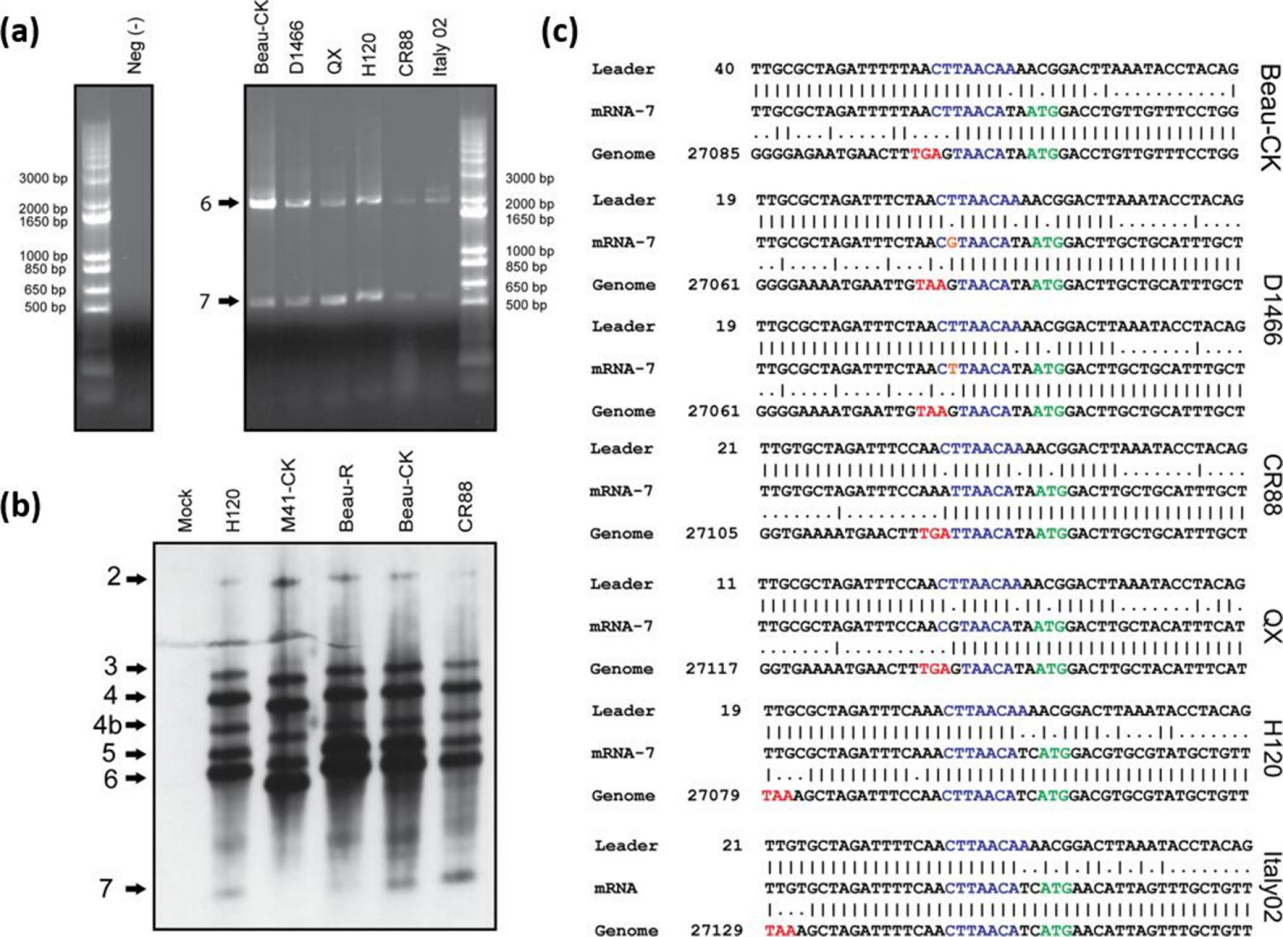
A gene 7 transcript is produced by diverse strains of IBV. (a) Leader-body PCR analysis of infected CK cell whole-cell lysate. Amplification of sgmRNAs by PCR and subsequent separation by agarose-gel electrophoresis produces two bands in the instance of Beau-CK, D1466, QX, H120 and CR88, and three bands in the instance of Italy-02. All six viruses produce bands corresponding to gene 6 (N protein,~2000 bp) and gene 7 (~500–600 bp). The larger band in Italy-02 relates to gene 5. (b) Northern blot analysis of infected whole-cell lysate. A clearly distinguishable band is present at the bottom of the gel for H120, Beau-R, Beau-CK and CR88 that corresponds to sgmRNA 7. Confirmatory to Fig. 1(b), M41-CK does not have a band corresponding with sgmRNA 7. All five viruses have bands corresponding to the other sgmRNAs as anticipated. However, there is a consistent pattern of bands between genes 6 and 7 across the IBVs that cannot be explained. (c) Sequence analysis of leader-body PCR products. All sequences are indicative of sgmRNAs; however, the TRS-B and the site of leader-body switch is not consistent. Genome positions for each IBV strain are indicated on the left (NCBI GenBank accession numbers: AJ311317, MN548285, MN548286, MN548287, MN548288, MN548289). Beau-CK and QX both have a partial match to the canonical 8 nucleotide TRS like Beau-R (TAACA). CR88 has a partial match of 6 nucleotides (TTAACA) versus the canonical TRS. Sequencing of D1466 resulted in a fluctuation (orange) as to where the leader-body junction for sgmRNA 7 is situated, but possess a TAACA TRS. H120 and Italy-02 possess the canonical 8 nucleotide TRS. The distance between the N stop codon (red) and the TRS for gene 7 is longer in H120 and Italy-02 versus the other studied IBVs. Red, N stop codon; blue, nucleotide matches to the canonical TRS; green, prospective ORF 7 start codon; orange, single nucleotide difference in D1466 leader/body junction.

Investigation of a variety of both field and vaccine IBV strains by leader-body junction RT-PCR and Northern blot analysis identified sgmRNA 7 in several other viruses (Fig. 2a, b), including the vaccine strains H120 and CR88 and pathogenic strains D1466, Italy-02 and QX. Leader-body junction RT-PCR followed by sequence analysis demonstrated that there is variability in the number of nucleotides that constitute the TRS-B (Fig. 2c). Both the genomes of Beau-CK and QX contain a TRS-B of UAACA, which aligns with nucleotides 3 to 7 of the TRS-L, CUUAACAA. This also confirms the molecular clone Beau-R is comparable to the parental virus Beau-CK (Figs 1c and 2c). Unlike the Beaudette strains, the TRS-B for CR88 reads UUAACA aligning to positions 2 to 7 of the TRS-L, and the TRS-B in the genomes of Italy-02 and H120 have the highest sequence homology with the TRS-L, differing only at position 8. Interestingly, both H120 and Italy-02 have leader-like sequence located immediately upstream of the TRS-B, resulting in a greater number of nucleotides between the N stop codon and the TRS-B than the other strains assessed. The pathogenic strain D1466 also presents a more complex scenario as in this assay two populations of sgmRNA 7 were identified. One population contains a TRS of CUUAACA, which similarly to Beaudette had nucleotides 3–7 matching the TRS-B. The other population contained a TRS of CGUAACA, differing at position 2, with this nucleotide appearing to be derived from the genome sequence at position 27 079, rather than the leader sequence at position 37. In the latter population, it appears that the recombination event between TRS-L and TRS-B has occurred between positions 1 and 2 of the TRS-L, with position 1 of the TRS derived from TRS-L and positions 2–7 from the genome, TRS-B (Fig. 2c). Despite the variation, all the TRSs for the sgmRNA 7, in all the IBV strains assessed, possessed a common core sequence (CS) of UAACA, supporting the notion that the TRS-B is universally UAACA, and also suggesting this could be the minimum requirement for effective transcription.

The leader-body junction analysis was initially carried out using universal primers with RNA extracted from infected CK cells. To ensure there was no RT-PCR/priming error, primers were re-designed to be strain specific (Table 2). Intracellular RNA was then extracted from infected CK cells, embryonated eggs and TOCs and the leader-body junction RT-PCRs were carried out utilizing the strain-specific primers. The results of this analysis matched those results detailed in Fig. 2; therefore, demonstrating that the sgmRNA was not an artefact of virus replication in cell culture (data not shown).

**Table 2.** Quantifying transcript sequence origin for each sgmRNA in Beau-CK pear-merged reads possessing a leader sequence were subsequently screened for the presence of a TRS. The 5′ end of the read (relative to the genome) up to and including the leader sequence was removed from the read and the following 8 nucleotide sequence (matching the length of the canonical IBV TRS) was captured. The number of identical 8 nucleotide sequences for each gene was counted with instances occurring at a frequency >1 % reported. In gene 2 (S protein), for example, 53.4 % of transcripts possessed a CTTAACAA sequence, whereas 45.5 % possessed a CTGAACAA sequence. The difference at position 3 presumably originates from an inconsistent switch point from body to leader as the TRS-L possesses a T and genome sequence possesses a G at this site.

sgmRNA (products)	TRS-L	Genome	Transcript sequence	Instances (percentage)
2 (S)	CTTAACAA	CTGAACAA	CTTAACAA CTGAACAA	3240 (53.4 %) 2754 (45.4 %)
2*	CTTAACAA	CCAAACAA	CTTAACAA CTAAACAA	398 (90.7 %) 35 (8.0 %)
3 (3a/3b/E)	CTTAACAA	CTGAACAA	CTTAACAA CTGAACAA	1777 (61.5 %) 1099 (38.0 %)
4 (M)	CTTAACAA	CTTAACAA	CTTAACAA	4148 (99.0 %)
4b (4b/4 c)	CTTAACAA	GTGACCAA	CTTAACAA CTTACCAA	922 (96.0 %) 19 (2.0 %)
5 (5a/5b)	CTTAACAA	CTTAACAAAAACTTAACAA	CTTAACTTTTTCTTAACAA -----------CTTAACAA	400 (3.0 %) 12 500 (95.2 %)
6 (N)	CTTAACAA	CTTAACAA	CTTAACAA	35 826 (99.7 %)
7 (7)	CTTAACAA	AGTAACAT	CTTAACAT CGTAACAT	90 (82.6 %) 18 (16.5 %)

**Fig. 3.**
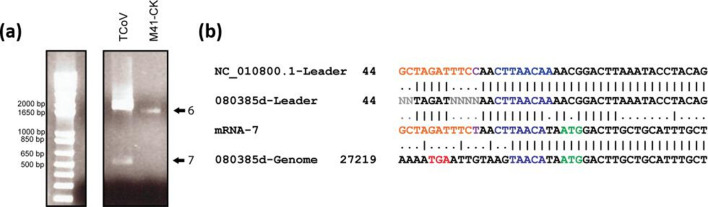
A gene 7 transcript is also present in TCoV. (a) Leader-body PCR analysis of European TCoV strain 080385d RNA harvested from infected turkey embryos. Separation of PCR-amplified TCoV sgmRNAs by agarose gel electrophoresis produces a ~2000 bp band and a~500–600 bp band corresponding to the expected sizes of gene 6 and gene 7, respectively. The gene 7 band has notably reduced intensity versus gene 6 as seen in IBV. (b) Sequencing of leader-body PCR products. The TCoV 080385d sequence is available (NCBI GenBank accession number KR822424.1); however, the 5′ extremities including the leader sequence have not been fully resolved. A sequence comparison versus the TCoV strain MG10 RefSeq (NCBI GenBank accession number NC_010800.1) confirms the smaller band matches the 5′ leader TCoV sequence (orange) with this exception of 1 nucleotide (purple). Reads confirm that TCoV 080385d gene 7 utilizes a TAACA CS. Red, N stop codon; blue, matches to the canonical CS (CTTAACA); green, prospective ORF 7 start codon; grey, ambiguity code (Ns) in sequence; orange, nucleotide matches between TCoV 080385d gene 7 leader sequence and TCoV MG10 NCBI RefSeq (NCBI GenBank accession number NC_010800.1); purple, nucleotide mismatches between 080385d gene 7 leader sequence and TCoV MG10 NCBI RefSeq NC_010800.1.

To determine whether the presence of sgmRNA 7 is potentially common to other gammacoronaviruses, its presence in another avian *Gammacoronavirus*, TCoV, was investigated. Total RNA was extracted from TCoV-infected turkey embryos and leader-body junction RT-PCR analysis was carried out to confirm the presence of a PCR product that related to a sgmRNA located at the 3′ of the genome, after the N gene (Fig. 3). Sequence analysis of the TCoV-derived PCR products identified a PCR product that was analogous to the IBV sgmRNA 7, which contained a leader sequence fused to the 3′ end of the TCoV genome via a TRS-B of UAACA.

### Determination of the origin of the TRS of the newly identified sgmRNA

Two rIBVs were previously constructed in the investigation of the sgmRNA 4b: BeauR-l-CTGAACAA, which has a U→G point mutation at position 3 in the TRS-L, and BeauR-l-CTTAACAT that has the mutation A→U at position 8 [6]. Results from the previous work, using the rIBVs, demonstrated that in the case of gene 4b, positions 1–5 of the TRS were derived from the TRS-L and positions 6–8 of the TRS were derived from the TRS-B, with the template switch during sgmRNA synthesis occurring between positions 5 and 6.

**Fig. 4.**
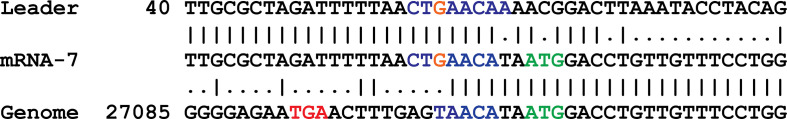
Template switch between the TRS-L and TRS-B occurs between positions 3 and 4 of the TRS-L. Sequence analysis of sgmRNA 7 transcripts expressed in CK cells infected with rIBV BeauR-l-CTGAACAA. The virus genome sequence is shown at the bottom, the sequence of sgmRNA 7 in the middle, and the virus genome leader sequence at the top. TRSs are coloured in blue, with the mutated nucleotide at position 3 of the TRS-L coloured in orange. The stop codon for N is highlighted in red and the start codon for ORF 7 in green. The sgmRNA 7 includes the G at position 3, which has originated from the leader sequence, indicating that the template switch has occurred between positions 3 and 4 of the TRS-L. Genome positions according to NCBI GenBank accession number AJ311317 are indicated on the left.

Leader-body junction analysis on RNA extracted from BeauR-l-CTGAACAA infected CK cells for characterization of sgmRNA 7 identified the transcript possessed a CUGAACU sequence (Fig. 4), confirming that the template switch included the TRS-L mutation. This would indicate that the template switch occurs after position 3 on the canonical TRS. Unfortunately, BeauR-l-CTTAACAT could not be used in this study as the nucleotide in position 8 in the TRS-B for the sgmRNA 7 is already a U.

### Potential protein encoded by the novel ORF

**Fig. 5.**
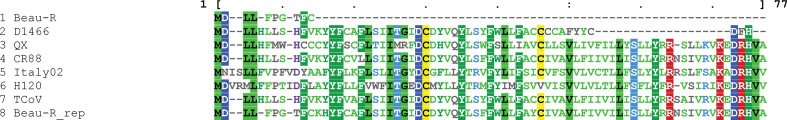
Alignment of ORF 7 prospective proteins in different IBV strains and TCoV using MView. Gene 7 sgmRNA sequences were screened for the presence of an AUG start codon. A 53–77 aa protein sequence was identified in TCoV and all IBVs with exception to Beau-R, which has a highly truncated 10 aa protein sequence. A single T deletion in Beau-R at position 27 141 (BeauR-ORF7rep) results in a frameshift mutation and the potential to generate a 73 aa product.

Analysis of the genomes from the IBV strains investigated, as well as TCoV, demonstrated a large degree of variability in the predicted protein sequences of 73 aa associated with sgmRNA 7 (Fig. 5). However, for both Beaudette and D1466, the predicted proteins are truncated when compared to the putative proteins from the other IBVs and TCoV. The Beaudette-encoded peptide consists of 10 aa and the D1466-encoded peptide of 53 aa. As previously mentioned, the genome of M41 does not encode sgmRNA 7 due to a deletion in this part of the genome (Fig. 1). Therefore, it is reasonable to assume that any potential protein encoded by sgmRNA 7 is non-essential for virus replication. Conversely, it is possible that expression of the protein may be detrimental to virus proliferation in cell culture considering that Beaudette, a virus which has been passaged over 300 times in various systems, has mutated to result in a severely prematurely terminated polypeptide.

**Fig. 6.**
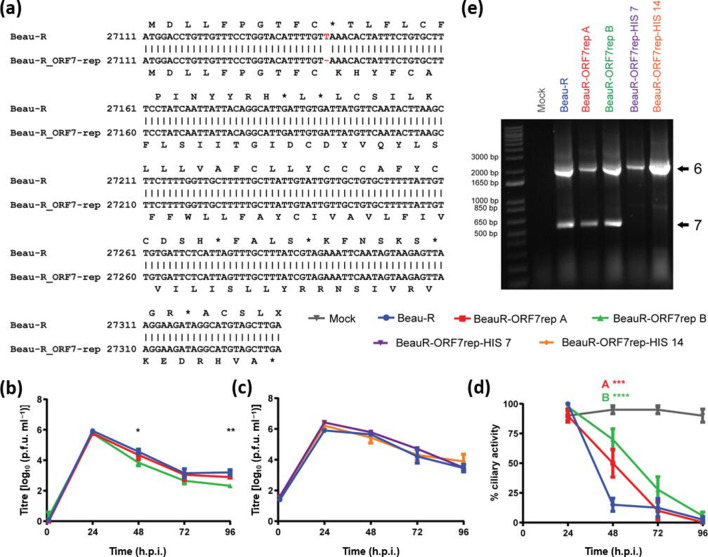
Repairing ORF 7 in Beau-R does not affect viral growth *in vitro*. (a) Pairwise alignment between Beau-R and BeauR-ORF7rep recombinant virus sequences. A single T deletion in Beau-R at position 27 141 (BeauR-ORF7rep) results in a frameshift mutation and the potential to generate a 73 aa product. Genome positions according to NCBI GenBank accession number AJ311317 are indicated on the left. (b, c) Growth kinetics of Beau-R, BeauR-ORF7rep and BeauR-ORF7rep-HIS viruses *in vitro*. Confluent CK cells seeded in six-well plates were infected with (b) 1×10^4^ p.f.u. or (c) 1×10^5^ p.f.u. At 1, 24, 48, 72 and 96 h.p.i., supernatant was harvested and assessed for infectious viral progeny by titration in CK cells. Each value represents the mean of three replicates with error bars representing sem. Statistical differences denoted by either * (*P*<0.05) or ** (*P*<0.005) were assessed using a two-way ANOVA paired with a Tukey test for multiple comparisons. The only differences identified were between Beau-R and BeauR-ORF7rep B at 48 and 96 h.p.i. (d) Ciliary activity in *ex vivo* TOCs. In replicates of ten, *ex vivo* TOCs were infected with 1×10^5^ p.f.u. of either Beau-R, BeauR-ORF7rep A or BeauR-ORF7rep B, or mock infected with medium. Ciliary activity was assessed by light microscope at 24, 48, 72 and 96 h.p.i. Each value represents the mean score of ten replicates with error bars representing sem. Statistical differences were assessed using a two-way ANOVA paired with a Tukey test for multiple comparisons. All three rIBVs were statistically different to mock infected from 24 to 96 h.p.i. (*P*<0.05). Statistical differences between Beau-R and BeauR-ORF7rep A and Beau-R and BeauR-ORF7rep B are highlighted by *** (*P*<0.0005) and **** (*P*<0.0001), respectively. No other statistical differences between the rIBVs at any time point were identified. (e) Leader-body PCR analysis of virus-infected or mock-infected CK cell whole-cell lysate. Amplification of sgmRNAs by PCR utilizing primer leader 1 and 93/100 produces two bands after separation by agarose gel electrophoresis. The larger sized product (~2000 bp) corresponds to the expected band size for gene 6 (N protein) transcripts and the smaller product (~500–600 bp) for gene 7 transcripts.

Using a reverse genetics system previously described by Casais *et al.* [20], a single nucleotide was deleted in Beau-R in order to cause a frameshift removing the associated premature stop codon (Fig. 6a). Two rIBVs were produced from independent homologous recombination events and independent rescue events, denoted BeauR-ORF7rep A and BeauR-ORF7rep B, both of which have the potential to express a full-length protein of 73 aa (Fig. 5) from sgmRNA 7. In an effort to detect this protein, two further viruses were generated, similarly from independent lineages, BeauR-ORF7rep-HIS 7 and BeauR-ORF7rep-HIS 14, both of which contain a His-Tag comprised of six histidine residues located at the N-terminal end of the repaired polypeptide. The 5′ end of ORF 7 was chosen for the His-tag modification instead of the 3′ end due to the proximity of the latter to the secondary RNA structures present in the 3′-UTR that regulate RNA synthesis [45–48].

The growth kinetics of both isolates of BeauR-ORF7rep and BeauR-ORF7rep-HIS were analysed in CK cells, and were found to be comparable to Beau-R (Fig. 6b, c). Of note, the titres for BeauR-ORF7-rep B were lower than Beau-R at both the 48 and 96 h.p.i. time points (*P*<0.05; Fig. 6b), although the difference in titres was minimal. *Ex vivo* TOCs were infected with BeauR-ORF7rep A and BeauR-ORF7rep B, and the ciliary activities assessed over a 96 h period in 24 h intervals (Fig. 6d). Both isolates did cause ciliostasis similar to the parental virus; however, the process appeared to be delayed with lower ciliary activities observed for Beau-R in comparison to the two rIBVs at 48 h.p.i. (*P*<0.005) (Fig. 6d). The observation that both rIBVs could induce ciliostasis alongside the growth kinetic assays completed in CK cells indicated that any potential protein encoded by ORF 7 is not detrimental to viral replication *in vitro* nor in *ex vivo* TOCs.

To determine whether both isolates of BeauR-ORF7rep and BeauR-ORF7rep-HIS produced modified sgmRNA 7 s for potential expression of the ORF 7 protein or His-tagged version, total RNA was extracted from infected CK cell lysate. Leader-body junction RT-PCR analysis confirmed that BeauR-ORF7rep expressed the modified sgmRNA 7; however, BeauR-ORF7rep-HIS did not (Fig. 6e). Sanger sequence analysis of an RT-PCR product to analyse the modified genome, utilizing RNA from the same cell lysate, demonstrated that the genome of the BeauR-ORF7rep-HIS virus retained the His-Tag modification located at the 5′ end of ORF 7. The results of the leader-body junction RT-PCR analysis indicate that the His-Tag modification appears to prevent the expression of the sgmRNA 7, indicating a possible role for sequences flanking the TRS-B in the regulation of sgmRNA expression.

### Investigation of a non-canonically transcribed sgmRNA within gene 2

**Fig. 7.**
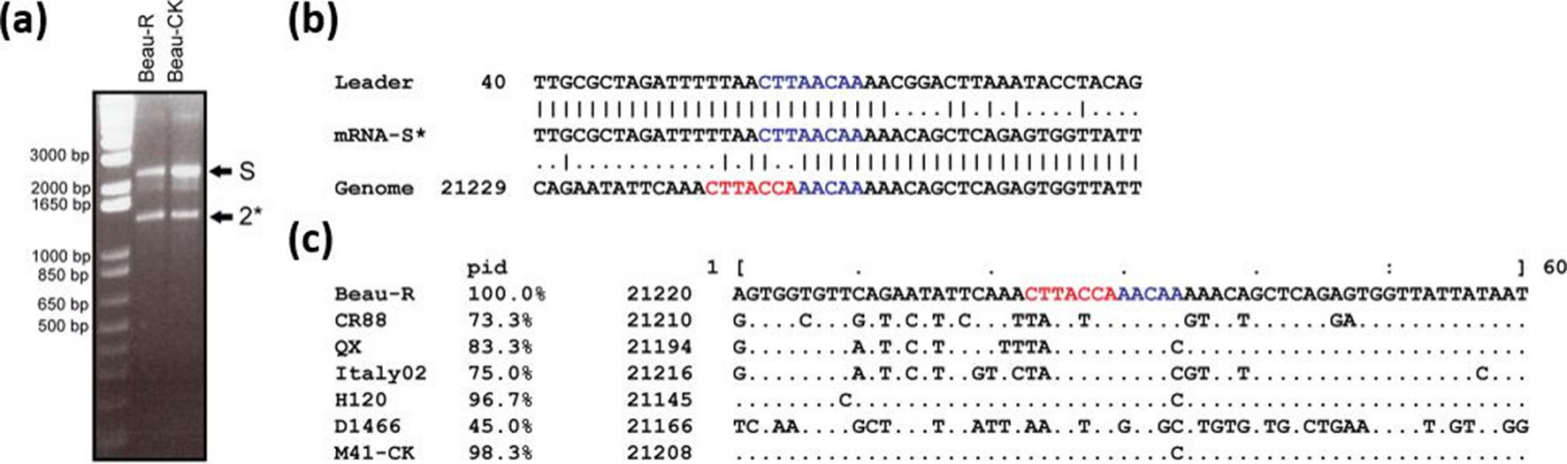
Detection of a transcript-like sequence between genes 2 and 3. (a) Leader-body PCR analysis of Beau-R and Beau-CK infected CK cell whole-cell lysate. Amplification of sgmRNAs by PCR utilizing primer leader 1 and BG139 produces two bands, one relating to gene 2 (S) transcripts and the second sgmRNA 2* transcripts. (b) Sequence analysis of leader-body PCR products identifies a TRS-B of AACAA. TRSs present in the leader, sgmRNA and the genome are highlighted in blue. Highlighted in red, upstream of the genomic TRS-B, is a TRS-like sequence. (c) Genome alignment of Beau-R (AY311317.1) position 21 220 to 21 279 surrounding the TRS-B (highlighted in blue) with other IBV strains. A TRS-like sequence is highlighted in red and percentage identity (pid) is indicated for each sequence compared to Beau-R. Genome positions for each IBV strain are indicated on the left (NCBI GenBank accession numbers: AJ311317, MN548285, MN548286, MN548287, MN548288, MN548289).

Previous work involving Northern blot analysis of CK cells infected with the rIBV BeauR-Δ-IR revealed the presence of an additional RNA species, denoted 2*, that related to a putative sgmRNA site located within gene 2 (S) [6]. An investigation, utilizing leader-body junction RT-PCR analysis of RNA extracted from Beau-R and Beau-CK infected CK cells, identified an RT-PCR product whose sequence contained the IBV leader sequence fused to the IBV genome within the S gene, at position 21 249 of the genome, via a non-canonical TRS-B of AACAA accounting for positions 4 to 8 of the TRS-L (Fig. 7a, b). Interestingly the sequence analysis also identified a TRS-like sequence of CUUACCA located immediately adjacent to the TRS-B (Fig. 7b).

To establish whether other IBV strains were capable of expressing sgmRNA 2*, total RNA was extracted from CR88, QX, Italy-02, H120, D1466 and M41-CK infected CK cell lysates and investigated by leader-body junction RT-PCR analysis. No RT-PCR product relating to Beaudette sgmRNA 2* was identified for any of the other IBV strains (data not shown). Sequence alignment of the IBV genomes, corresponding to the Beaudette sgmRNA 2* region, showed that all of the other IBV strains investigated, except for CR88, did not possess a TRS-B of AACAA (Fig. 7c), possibly accounting for the lack of sgmRNA 2* expression. Notably, all the other strains, except CR88, contained an A to C substitution at position 5 of the Beaudette TRS-B, which could suggest that a TRS-B of AACA is not sufficient to enable synthesis of sgmRNA 2*. Interestingly, CR88 does contain a TRS-B of AACAA, comparable to the Beaudette strain; however, despite several attempts by both leader-body junction RT-PCR analysis and Northern blot analysis an RNA relating to sgmRNA 2* could not be identified in CR88 infected cells. Further analysis of the genome sequence highlights that whilst CR88 and Beau-R share identical TRS-B sequences, there are nucleotide differences located in both the immediate 5′ and 3′ flanking regions, which may be responsible for why CR88 does not produce a sgmRNA 2*.

### Detection of Beau-CK transcripts in infected allantoic fluid

**Fig. 8.**
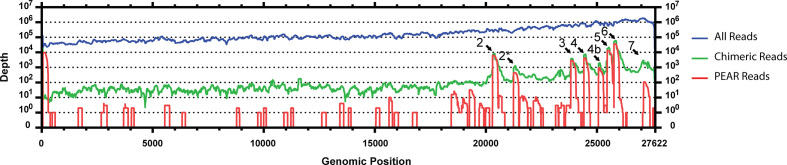
HiSeq analysis confirms the presence of genes 2* and 7, and indicates potential for other transcripts across the genome. Sequencing coverage of Beau-CK HiSeq reads to Beau-CK genome using three separate inputs. All reads (passing quality filter) were aligned to the Beau-CK using BBMap (blue). Beau-CK reads were also aligned the Beau-CK genome using bwa with reads flagged as chimeric subsequently aligned using BBMap (green). Forward and reverse reads were merged using pear and reads possessing the leader sequence selected. The 5′ end of the read (relative to the genome) up to and including the leader sequence was removed from the read, and the remainder aligned to the genome using bwa (red). The trace for the chimeric read coverage plot (green) indicate positions in the genome covered by reads that align in two places, whereas the trace in the leader-matching (red) coverage plot indicates positions that are covered by reads that also possess the leader sequence. Coverage peaks occur at sites of known transcripts (2, 3, 4, 4b, 5 and 6), with additional peaks corresponding to gene 2* and 7 transcripts also identifiable.

The identification of up to three non-canonically transcribed sgmRNAs, 2*, 4b and 7 from IBV, highlighted the possibility for IBV to generate a larger number of yet unidentified gene products. In an effort to investigate whether there is potential for even more sgmRNAs, a HiSeq dataset was generated to screen for the presence of additional putative sgmRNAs. The HiSeq dataset was generated by HiSeq analysis of allantoic fluid harvested from Beau-CK infected SPF embryonated hens' eggs. A total of 185 992 reads aligning to two distinct, non-consecutive positions of the genome (termed chimeric reads) were identified. Separately, a total of 73 846 reads possessing the IBV 5′ leader sequence were identified after merging quality-filtered reads using pear [42]. Peaks in coverage for both chimeric-mapped reads and leader-possessing reads correspond to sgmRNAs, with 2 (S), 3, 4 (M), 5 and 6 (N) clearly identified. Peaks corresponding to the non-canonically transcribed sgmRNAs 2*, 4b and 7 were also identified (Fig. 8). Additional peaks relating to either/both chimeric reads and reads containing the IBV 5′ leader sequence across the Beaudette genome, including some originating within gene 1, indicate the potential for previously unidentified sgmRNAs and putative associated proteins.

A second analysis was performed on the HiSeq dataset assessing the usage of specific TRSs for the known sgmRNAs (Table 2). This assessment demonstrated there is fluidity in the recombination events between the TRS-Bs and TRS-L, with variability in the recombination locations identified. The Beaudette sgmRNA 2 (S gene) TRS-B has the sequence CUGAACAA, meaning there is a mismatch at position 3 with the TRS-L, CUUAACAA. HiSeq analysis has shown that approximately 50 % of the chimeric reads identified a sgmRNA 2 possessing a TRS containing a G at position 3 and 50 % possess a U. This indicates there is the potential for variability in the location of the recombination event between two TRSs, with the recombination event between the S gene TRS-B and TRS-L occurring between positions 1 and 2 or 2 and 3 of TRS-B to include the G at position 3 in sgmRNA 2. In order to allow the U nucleotide at position 3, recombination would need to occur at a point somewhere between positions 3 to 8 of TRS-B. A similar occurrence was observed, from the HiSeq data, for sgmRNA 3 (gene 3), which has a TRS-B of CUGAACAA; approximately 60 % of the sgmRNAs contain a U at position 3 and ~40 % a G.

The sgmRNAs that utilize non-canonical TRS-Bs also demonstrate variable locations of the recombination events between the associated TRS-B and TRS-L; however, these also show a clear preference. For sgmRNA 4b (gene 4b) the genome reads GUCACCAA with the TRS-B identified as CAA. HiSeq analysis showed that the majority of sgmRNA 4b contained a TRS of CUUAACAA, but there is a very small population,~2 %, that contain CUUACCAA, a difference at nucleotide position 5. A similar situation was identified with sgmRNA 7,~80 % of sgmRNA-derived reads contained a TRS of CUUAACAU and~16 % contained CGUAACAU; the TRS-B has been identified to be UAACA. This observed plasticity in the location of the recombination event observed between the TRS-L and a TRS-B suggests that the presence of a fixed sequence TRS-B is not sufficient alone to regulate transcription, and that other regulatory factors are involved.

## Discussion

This work has identified and investigated additional examples of sgmRNAs transcribed from non-canonical TRS-Bs within the gammacoronavirus IBV genome, and further highlights the potential for coronavirus genomes to encode additional ORFs. Interestingly, whilst sgmRNA 2* and associated ORF 2*, are likely to be Beaudette-specific and not a shared feature of IBV strains, a similarly located ORF has been identified in SARS-CoV and in porcine respiratory coronavirus [12, 49, 50].

This report identifies another non-canonically transcribed sgmRNA, which like sgmRNA 4b, is seemingly universally shared between IBV strains. This sgmRNA, designated sgmRNA 7, is transcribed from a non-canonical TRS-B, UAACA, and contains a putative ORF located at the 3′ end of the genome immediately downstream of the N gene and upstream of the 3′-UTR: an area previously reported not to encode an ORF due to the lack of apparent TRS-B [51]. As this paper was in preparation, two further publications have identified this sgmRNA in the Beaudette strain of IBV, transcribed from a TRS-B of UAACA or a putative TRS-B of UAACAU [7, 14]. However, this report furthers the findings of both Dinan *et al.* [7] and An *et al.* [14] by (i) confirming the TRS-B of UAACA in the Beaudette strain and (ii) identifying the presence of sgmRNA 7 and associated ORF in several other IBV strains, both vaccine and field isolates, including CR88 and QX, respectively. Interestingly, there is variability in the sequence of the TRS-B between the strains of IBV, with the genome of CR88 containing a 6 nucleotide TRS-B of UUAACA and Italy-02 a 7 nucleotide TRS-B of CUUAACA. Of particular note, An *et al.* [14] stated that H120 did not express sgmRNA 7 due to the lack of a TRS-B of UAACA; however, this report has demonstrated that H120 does transcribe sgmRNA 7, but utilizes an extended TRS-B of 7 nucleotides, CUUAACA. The TRS-B is, therefore, only 1 nucleotide short of being considered a canonical TRS-B and, interestingly, immediately upstream of the TRS-B is a leader-like sequence, also observed in the Italy-02 genome. We also demonstrated that the M41-CK strain does not encode sgmRNA 7 due to a 185 nucleotide deletion that removes the 3′ terminal CA of the TRS-B, as well as the majority of the genome sequence encoding the associated ORF.

**Fig. 9.**
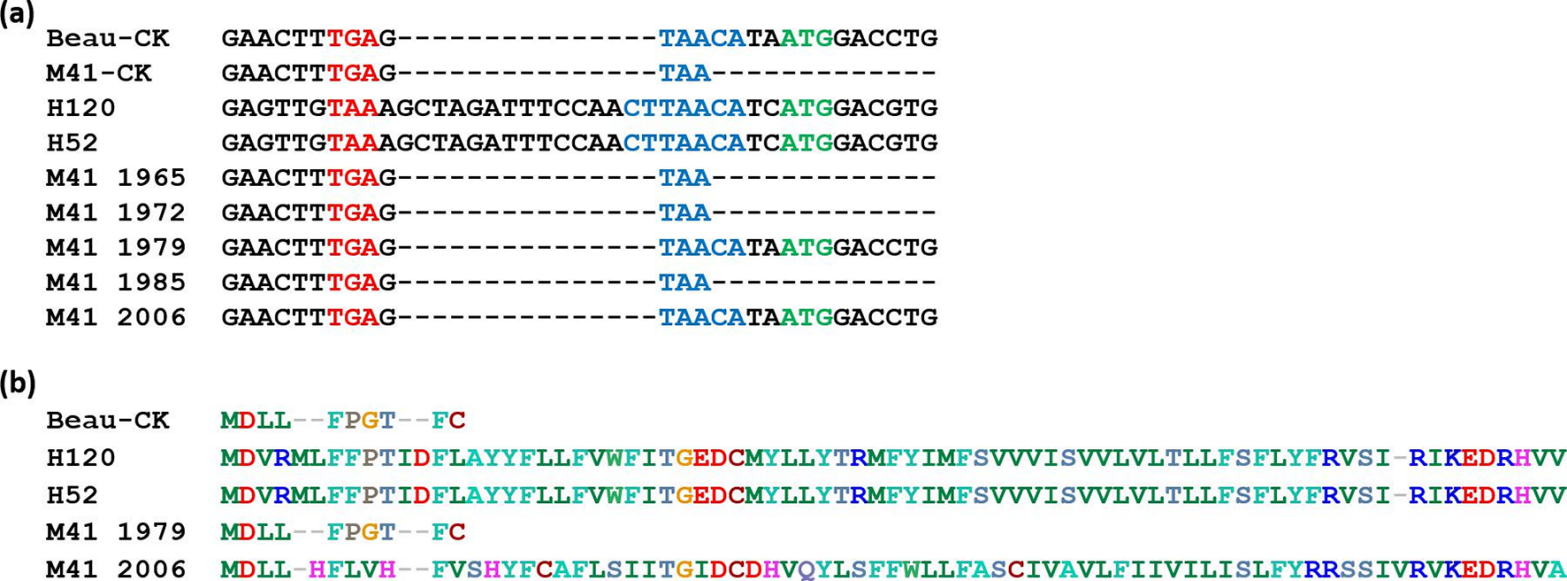
The presence of gene 7 varies between strains of the Massachusetts serotype. (a) The nucleotide sequences of different Mass strains were compared over the start of gene 7. Red, N stop codon; blue, nucleotide matches to the canonical TRS; green, prospective ORF 7 start codon. (b) A comparison of the amino acid sequences. NCBI GenBank accession numbers: AJ311317.1, MK728875.1, FJ904720.1, FJ904721, FJ904722, FJ904723.1, FJ904713, EU817497.1 and MN548287.

Further comparison of other Mass serotype strains indicates that there is some variability between isolates. Whilst vaccine strains H52 and H120 share an identical ORF 7 sequence (Fig. 9), other Mass isolates differ in this region. Identical to the Beaudette strain, a Mass isolate from 1979 (NCBI GenBank accession number FJ904722) contains a single nucleotide insertion within ORF 7, which results in a premature stop codon truncating the polypeptide to 10 aa. Mass isolates from 1965, 1972 and 1985 (NCBI GenBank accession numbers FJ904720.1, FJ904721 and FJ904723.1) possess a shortened TRS of TAA and do not have an AUG start codon, like the M41-CK strain used in this study (Fig. 9a). Interestingly, a Mass isolate from 2006 (NCBI GenBank accession number FJ904713) contains the TAACA TRS like Beau-CK, but encodes a different sgmRNA 7 sequence (Fig. 9b). All of these Mass isolates were isolated from broiler chickens with clinical signs of disease [52], so the variation in ORF 7 cannot be linked to pathogenicity.

In alpha*-* and betacoronaviruses, each TRS is thought to contain a CS that is conserved between the TRS-L and TRS-B. Flanking the CS is a number of nucleotides, located at the 5′ and 3′ ends of a TRS that varies between TRS-Bs [15, 53]. Research has shown that sequence homology between the CS-L and CS-B is proportional to the abundance of sgmRNA observed [15, 16]. In all the IBV strains assessed in this report, sgmRNA 7 would appear to have a CS of UAACA; however, this CS is not shared between all the known TRS-Bs in the genome. HiSeq data generated from Beau-CK infected allantoic fluid identified that only nucleotide A at position 4 and CA at positions 6 and 7, respectively, of the IBV TRS-L, CUUAACAA, are shared (Table 2). As a note, the TRS-B of sgmRNA 4b only contains CAA, position 6 to 8 of the TRS-L [54], but there is an A nucleotide located at position 4 that corresponds with both TRS-L and the body of the genome. What is interesting regarding sgmRNA 7 is that nucleotides CA at positions 6 and 7 of the TRS-L, CUUAACAA, are those that are missing from the TRS-B in the M41-CK genome. This perhaps indicates that these nucleotides are required for the expression of sgmRNA 7.

Regardless of the dynamics and regulation of transcription, there is precedent for a functional accessory gene to be located between the N gene and the 3′-UTR, with such genes extensively studied in the alphacoronaviruses transmissible gastroenteritis virus (TGEV) and feline infectious peritonitis virus (FIPV) [55–58], deletion of which does not affect replication *in vitro* but does affect pathogenicity *in vivo* [57, 59]. In FIPV, the sgmRNA 7 encodes two proteins 7a and 7b, 101aa/10 kDa and 207aa/~24 kDa, respectively. Similarly to TGEV, it has been demonstrated that the 7ab cluster is essential for virulence *in vivo*, but not always for replication *in vitro*, with deletion knock-out viruses able to replicate well in feline FCWF cells but not peripheral blood monocytes [56, 58, 60]. The function of sgmRNA 7 in IBV, and whether a protein is produced from the sgmRNA, remains undetermined. An *et al.* [14] reported that there was no AUG start codon present, and concluded that sgmRNA 7 was non-coding [14]. Our report contradicts these findings by identifying an AUG start codon present in all IBV strains investigated, except for some Mass viruses including M41-CK, located 2 nucleotides from the 3′ end of the TRS-B. This proximity of the TRS-B to the AUG start codon is unusually close; the distances between the TRS-B and the AUG in Beau-R for genes 2, 3, 4, 5 and 6 are 52, 23, 77, 20/6 and 93 nucleotides, respectively.

Dinan *et al.* [7] have also shown the IBV ORF 7 AUG is occupied by ribosomes on RNA isolated from Beau-R infected CK cells, indicating that sgmRNA 7 is translated [7]. Unfortunately, our efforts to His-Tag the full-length Beaudette ORF 7 protein were unsuccessful with the His-Tag sequence insertion disrupting the transcription of sgmRNA 7. Whilst this was disappointing, it has provided evidence of the importance of the flanking regions located at the 3′ end of this TRS-B. An *et al.* also demonstrated the importance of the 3′ flanking sequence, particularly the sequence AAUGG at position 27 110–27 114 [14]. Mutations of this motif to UUUCC completely disrupted sgmRNA 7 expression; although it must be noted these mutations also disrupted the start codon. The His-Tag sequence insertion detailed in this report also disrupts the AAUGG motif by trans-locating the G at position 27 114 to 27 132 of the genome. RNA modelling using the RNAstructure tools webserver version 6.0.1 (2018) [61] suggests a change in RNA structure as a consequence of the 18 nucleotide His-Tag sequence insertion. RNA structure has been shown to play a regulatory role in coronavirus sgmRNA synthesis and in particular the interaction of distally located motifs [53, 62, 63]. It is also likely that flanking regions surrounding the TRS-B and/or RNA structure play a role in the expression of sgmRNA 2*, as despite the presence of a TRS-B, CR88 does not express this sgmRNA, indicating that other required regulatory factors are not present or are not active within the genome.

To assess whether the IBV genome could potentially encode additional undefined ORFs, a HiSeq dataset generated from Beau-CK infected allantoic fluid was investigated. This analysis highlighted that there are several chimeric RNAs consisting of the IBV leader sequence fused to different regions throughout IBV genome, including within the replicase gene (gene 1), all representing potential sgmRNAs. Whether these sequences account for biologically relevant sgmRNAs remains an unanswered question; however, this is not the first report to identify additional chimeric reads through sequencing analysis [64, 65]. Of particular note is the investigation of the arterivirus simian haemorrhagic fever virus, which identified an additional 51 functional TRS-Bs, greatly increasing the number of potential ORFs, with a number of the newly identified TRS-Bs located within gene 1 [64]. Analysis of this dataset has also highlighted that the location of the recombination event between the TRS-L and TRS-B is not fixed, which could indicate for the role of secondary regulating factors.

In conclusion, this report has provided further evidence for the presence of an ORF transcribed from non-canonical TRS-B located between the N gene and 3′-UTR in several strains of the gammacoronavirus IBV, denoted ORF 7. Investigation of the newly identified sgmRNA, as well as the investigation of sgmRNA 2*, indicates roles of the 5′ and 3′ flanking sequences of the TRS-B in the transcription of sgmRNAs. The HiSeq data generated in the report also identifies that there is variation in the location of the recombination event between the TRS-L and TRS-B, and additionally highlights the possibility of the IBV genome encoding further ORFs likely transcribed at low frequency from non-canonical TRS-Bs. The coronavirus replication cycle is complex and not fully understood and it is, therefore, important for future research to investigate these novel ORFs transcribed from non-canonical TRS-B sequences and to assess their role in IBV replication.
